# Comparison of chest CT findings in nontuberculous mycobacterial diseases vs. *Mycobacterium tuberculosis* lung disease in HIV-negative patients with cavities

**DOI:** 10.1371/journal.pone.0174240

**Published:** 2017-03-27

**Authors:** Cherry Kim, So Hee Park, Sang Young Oh, Sung-Soo Kim, Kyung-Wook Jo, Tae Sun Shim, Mi Young Kim

**Affiliations:** 1 Department of Radiology and the Research Institute of Radiology, University of Ulsan College of Medicine, Asan Medical Center, Seoul, Korea; 2 Department of Radiology, Ansan Hospital, Korea University College of Medicine, Danwon-gu, Ansan-si, Gyeonggi, Korea; 3 Pulmonary and Critical Care Medicine, Kyung Hee University Hospital at Gangdong, Seoul, Korea; 4 Department of Healthcare Management, Cheongju University, Cheongju, Korea; 5 Division of pulmonary and Critical Care Medicine, University of Ulsan College of Medicine, Asan Medical Center, Seoul, Korea; National Institute of Infectious Diseases, JAPAN

## Abstract

**Objectives:**

This article focuses on the differences between CT findings of HIV-negative patients who have cavities with nontuberculous mycobacteria (NTM) disease and those with *Mycobacterium tuberculosis* infections (TB).

**Methods:**

We retrospectively reviewed 128 NTM disease patients (79 males and 49 females) with cavities in chest CT, matched for age and gender with 128 TB patients in the same period. Sputum cultures of all patients were positive for pathogens. Two independent chest radiologists evaluated the characteristics of the largest cavity and related factors.

**Results:**

Interobserver agreement was excellent (κ value, 0.853–0.938). Cavity walls in NTM disease were significantly thinner (6.9±4 mm vs 10.9±6 mm, P<0.001) and more even (the ratio of thickness, 2.6±1 vs 3.7±2, P<0.001) than those in TB. The thickening of adjacent pleura next to the cavity was also significantly thicker in NTM than TB (P<0.001). However, in the multivariate analysis, thickening of adjacent pleura was the only significant factor among the representative cavity findings (Odds ratio [OR], 6.49; P<0.001). In addition, ill-defined tree-in-bud nodules (OR, 8.82; P<0.001), number of non-cavitary nodules (≥10mm) (OR, 0.72; P = 0.003), and bronchiectasis in the RUL (OR, 5.3; P = 0.002) were significantly associated ancillary findings with NTM disease in the multivariate analysis.

**Conclusions:**

The major cavities in NTM disease generally have thinner and more even walls than those in TB. When cavities are associated with adjacent pleural thickening, ill-defined satellite tree-in-bud nodules, or fewer non-cavitary nodules ≥10 mm, these CT findings are highly suggestive of NTM disease rather than TB.

## Introduction

Nontuberculous mycobacteria (NTM) are ubiquitous in the environment and are increasingly being recognized as significant in chronic pulmonary infections [1]. The most frequently encountered NTM lung disease worldwide is caused by *Mycobacterium avium-intracellulare complex* (MAC) [2–4]. Although the incidence and prevalence of NTM disease vary between populations, both have tended to increase over time [4–9]. In South Korea as well, the isolation of NTM on both solid and liquid media, and NTM disease, have been increasing in clinical practice [10,11].

South Korea has an intermediate tuberculosis burden, and therefore it is important to distinguish NTM disease from *Mycobacterium tuberculosis* disease (TB). The evaluation of mycobacterial infections by sputum study, culture, and TB-PCR usually takes a long time, and a quicker way of discriminating the two diseases is necessary for prompt treatment. There are some differences in the conventional radiographic features between pulmonary NTM disease and TB. NTM disease tends to cause thin-walled cavities with less surrounding parenchymal infiltrate, to have less bronchogenic but more contiguous spread of disease, and produce more marked involvement of the pleura over the involved areas of the lungs on chest radiographs [1,12]. Thin section chest CT has shown that up to 90% of MAC patients with non-cavitary disease in the mid and lower lung fields have associated multifocal bronchiectasis, with many patients having clusters of small (<5 mm) nodules in the associated areas of lung [13–15]. However, cavities are common in both NTM disease [16] and TB [17] and are a hallmark of the active infectious state in both diseases [17,18].

To our knowledge, there are no previous studies that aimed to discriminate between the two diseases on the basis of CT of their representative cavities and associated ancillary findings. The CT characteristics of the cavities and associated ancillary findings might help clinicians and radiologists discriminate between the two disease entities and give prompt treatment to some patients. Therefore, the primary purpose of this study was to describe the differences between the CT findings of HIV-negative patients who have cavities with nontuberculous mycobacteria (NTM) disease and those with *Mycobacterium tuberculosis* infections (TB).

## Materials and methods

### Study population

This study was approved by the Institutional Review Board of our hospital (Approval number: 2016–0056), and the requirement for the patient informed consent was waived due to its retrospective manner.

The electronic database and the registry of mycobacterial infections (of T.S.S) of this academic tertiary referral institution were searched to identify NTM disease and TB patients proven by culture between November 2004 and December 2013. Finally, 177 NTM disease patients and 302 TB patients were identified. The former were diagnosed with NTM pulmonary infection and fulfilled the American Thoracic Society/Infectious Diseases Society of America criteria for the diagnosis of NTM disease [1].

The inclusion criteria for patients were as follows: a) at least one visible cavity lesion in CT (>10mm); b) TB or NTM proven by culture; c) CT images available before treatment; and d) >18-years-old. Subsequent exclusion criteria were as follows: a) poor image quality for evaluating lung lesions including cavities (n = 23); b) insufficient computerized medical records (n = 6); c) HIV-positive (n = 2); d) co-infection with other pathogens including invasive mold disease or pseudomonas (n = 16).

**Fig 1:**
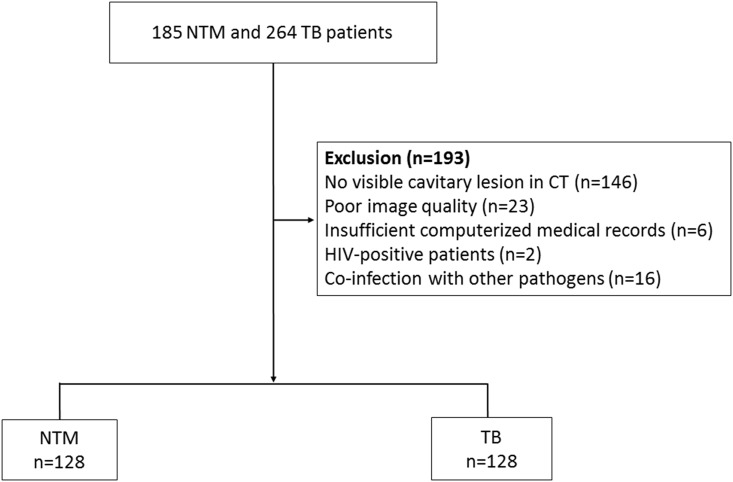
Flow chart of the study population. NTM = Nontuberculous mycobacterial, TB = *Mycobacterium tuberculosis* infection, CT = computed tomography, HIV = human immunodeficiency virus.

As a result, 128 NTM disease patients (79 males and 49 females) were finally identified. They were matched for age and gender with 128 TB patients in the same period. Fig 1 summarizes the patient selection process.

### CT imaging protocol

Various CT units were used over the >10-year follow-up period: a Sensation 16, SOMATOM Definition, SOMATOM Definition flash, SOMATOM Definition AS+ scanner (all manufactured by Siemens Medical Systems, Erlangen, Germany), LightSpeed 16, LightSpeed Plus, or LightSpeed VCT scanner (manufactured by GE Healthcare, Milwaukee, WI). The scanning parameters were as follows: beam collimation, 16 x 0.75 mm, 32 x 0.6 mm, or 64 x 0.6 mm; beam pitch, 0.984:1; gantry rotation time, 0.5 seconds; field of view to fit; 120 kVp. An automated dose reduction system was used (CARE Dose 4D, Siemens Medical Solutions; Auto mA/ Smart mA, GE Healthcare) with the maximum allowable tube current set at 100–400 mAs. Three- or five-mm-thick images at 3-mm/5-mm intervals without gaps were reconstructed in the axial planes. Coronal reformations have been routinely performed at a slice thickness of 5 mm for all CT scans since 2008. For contrast enhancement, intravenous contrast medium (100 cc of 300 mgI/mL nonionic contrast) was administered at a rate of 2–3 mL/seconds using an automatic power injector.

### CT interpretation

The CT scan nearest to the time of treatment, but before treatment, was selected for the radiologic review. The mean time between the first NTM and TB isolation and CT examination was 18.5±7.8 days (range, 0–34 days). These CT scans were retrospectively reviewed by 2 independent expert chest radiologists with 19 (M.Y.K) and 5 years (S.Y.O) of experience, respectively, blinded to the patient information, and patients were assigned in random order.

We evaluated the largest cavity in any of the six lobes of the lung (right upper lobe [RUL], right middle lobe [RML], right lower lobe [RLL], left upper lobe [LUL], lingular division, and left lower lobe [LLL]) as the main target lesion. The size of the cavity, the thickness of the cavity wall including the thinnest and thickest portions, the ratio of the thickest portion to the thinnest portion, and the prevalence of focal thickening of the pleura adjacent to the target cavity were evaluated in the lung window setting.

As ancillary findings we evaluated the size (mm, none, <3mm, 3-6mm, >6mm) and margins (ill-defined/well-defined) of the satellite branching centrilobular nodules (tree-in-bud pattern) surrounding the cavity, the presence of pleural effusion, number of non-cavitary nodules ≥10mm, the presence of bronchiectasis in any of the six lobes, and the presence of bronchiectasis involving more than 5 lobes.

### Review of other clinical data

One of the authors (S.H.P with clinical experience of three years) reviewed the medical charts. Age, gender, body mass index (BMI), smoking history, previous TB history, underlying disease (DM, the status of diabetes control, malignancy, and chronic obstructive pulmonary disease [COPD]), initial symptoms (cough, sputum, hemoptysis, dyspnea, or fever), and laboratory findings (albumin, white blood cell [WBC], C-reactive protein [CRP]) of all the study patients were recorded. Uncontrolled DM was defined as glycated hemoglobin (HbA1C) level ≤ 7.0% [19]. NTM pathogens proven in culture were also recorded.

### Statistical analysis

To compare clinical, demographic, and CT findings in the two study populations, Student’s t-test was used for continuous variables and the chi-square test for categorical variables. Variables were subjected to multivariate analysis by logistic regression and forward stepwise selection after univariate analysis. Interobserver variation was quantified by κ coefficients of agreement (κ values of 0.00–0.20, poor agreement; 0.21–0.40, fair agreement; 0.41–0.60, moderate agreement; 0.61–0.80, good agreement; and 0.81–1.00, excellent agreement). A P value < 0.05 was considered significant. All statistical analyses were performed with SPSS package, version 21.0 (SPSS, Chicago, Ill) by an expert statistician (S.S.K., 10 years’ experience).

## Results

### Comparison of the clinical characteristics of the two study populations

Clinical findings for the two study population are summarized in Table 1. The BMI of the NTM disease patients was lower than that of the TB patients (P<0.001). There were no significant differences in age and gender. Never smokers and patients with previous histories of TB were more common in the NTM disease group (P<0.001). DM and COPD were more common in TB (P<0.001 and P = 0.045, respectively). Among DM patients, the status of diabetes control was not significantly different between NTM and TB patients. The significant factors in multivariate analysis were never smoker (OR [odds ratio], 6.52; CI [confidence interval], 1.88–22.6; P = 0.003), previous TB history (OR, 3.57; CI, 1.23–10.4; P = 0.02), and dyspnea (OR, 0.05; CI, 0.003–0.73; P = 0.03). *M*. *intracellulare* was the most common pathogen in the NTM disease group (48.4%), followed by *M*. *avium* (30%), *M*. *abscessus* (11.7%), *M*. *kansasii* (6.3%), and others (3.9%) including *M*. *chimaera*, *M*. *szulgai*, *M*. *chelonae*, and *M*. *fortuitum*. The proportion of patients with a positive sputum AFB smear was similar between NTM and TB group (49.2% [63/128] vs. 46.1% [59/128], P = 0.532).

**Table 1:** Clinical characteristics of 256 patients with nontuberculous mycobacterial infections and *Mycobacterium tuberculosis* infections.

Clinical finding^a^		NTM (%) (n = 128)	TB (%) (n = 128)	P-value
**Age (years, mean±SD)**^b^		60.8±13.1	60.2±10.4	0.666
**Gender**				>0.999
	Male	79 (61.7)	79 (61.7)	
	Female	49 (38.3)	49 (38.3)	
**BMI**^b^		20.3±3.0	22.1±2.9	<0.001
**Smoking**				<0.001
	Current smoker	14 (10.9)	49 (38.3)	
	Ex-smoker	44 (34.4)	39 (30.5)	
	Never smoker	70 (54.7)	40 (31.3)	
**Previous PTB history**		62 (48.4)	17 (13.3)	<0.001
**Underlying disease**				
	DM	14 (10.9)	46 (35.9)	<0.001
	**Status of diabetes control**			0.535*
	• Well-controlled DM	7 (50)	63)	
	• Uncontrolled DM	7 (50)	17 (37)	
	Malignancy	9 (7.0)	17 (13.3)	0.098
	Chronic lung disease			
	COPD	12 (9.4)	23 (18)	<0.045
	ILD	11 (8.6)	5 (3.9)	0.121
**Symptom**				
	Cough	107 (83.6)	82(64.1)	<0.001
	Sputum	91 (71.1)	69(53.9)	0.005
	Hemoptysis	35 (27.3)	16(12.5)	0.003
	Dyspnea	1 (0.8)	19(14.8)	<0.001
	Fever	9 (7.0)	5(3.9)	0.272
**Laboratory finding**^b^				
	Albumin (g/dL, mean±SD)	3.7±0.6	3.5±0.6	0.091
	White blood cell (uL, mean±SD)	6880±2068	7365±2209	0.074
	C-reactive protein (mg/dL, mean±SD)	1.6±2.0	2.4±3.3	0.051

NTM = Nontuberculous mycobacteria, TB = *Mycobacterium tuberculosis* infection, SD = standard deviation, BMI = body mass index, COPD = chronic obstructive pulmonary disease, ILD = interstitial lung disease.

*Significance within DM patients, not in the whole study population

^a^ chi-square test

^b^ Student’s t-test

### Comparison of the CT findings in the study populations

**Fig 2:**
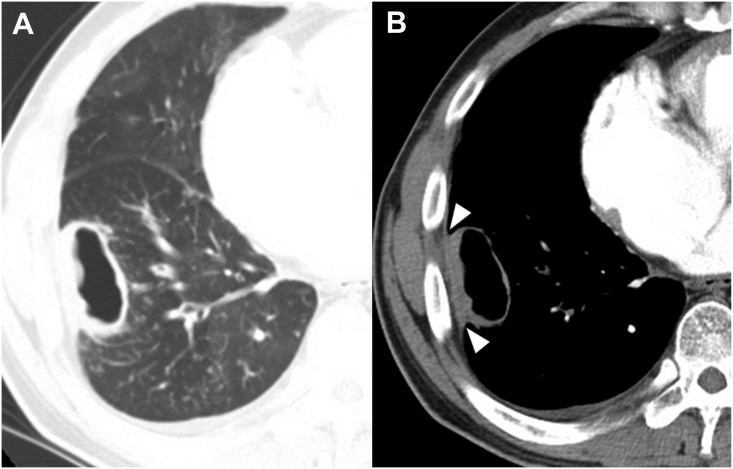
CT images of a *Mycobacterium avium* pulmonary infection in a 44-year-old man. Axial CT images with lung and mediastinal window settings (2.5-mm slice) were obtained at the level of the segmental bronchi of the right lower lobe. The CT images show relatively thin and relatively even thickening of the cavity, and tiny ill-defined satellite nodules (<4 mm) with air trapping in the right lower lobe. Note pleural thickening next to the cavity (**B**) (arrowheads).

**Fig 3:**
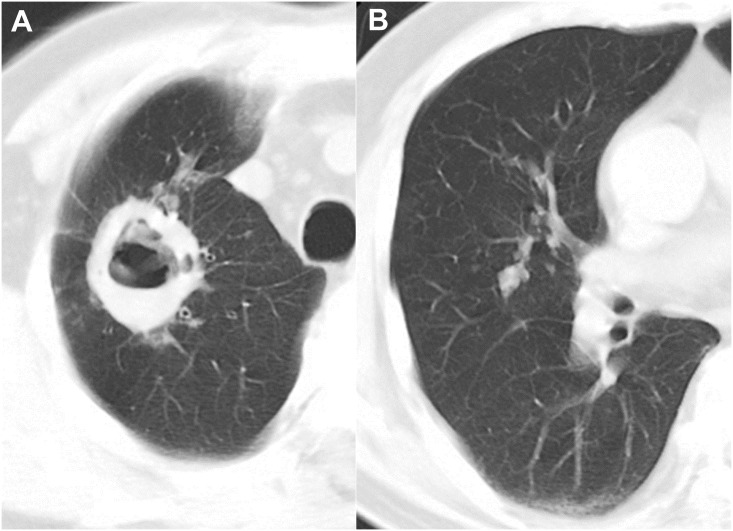
CT images of *Mycobacterium tuberculosis* infection in a 67-year-old man. Chest CT axial images with a lung window settings (2.5-mm slice) were obtained at the levels of the superior vena cava and right middle lobar bronchus. The CT image show a cavity with a thick and irregular wall and multiple satellite tree-in-bud nodules in the right upper lobe (**A**). Note the multiple well-defined tree-in-bud nodules (>6 mm) in the right middle lobe (**B**).

**Fig 4:**
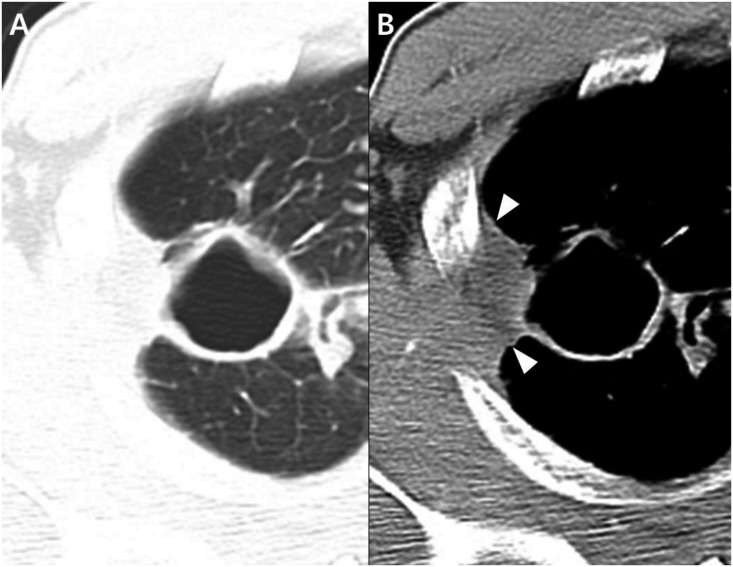
CT images of a *Mycobacterium intracellulare* pulmonary infection in a 41-year-old woman. Chest CT axial images with lung and mediastinal window settings (2.5-mm slice) show a thin-walled cavity (**A**) with adjacent pleural thickening (**B**) (arrowheads).

Interobserver agreement between the two readers was excellent (κ value, 0.853–0.938). The CT findings are presented in Table 2. In the analysis of the largest cavities, both the thickest and the thinnest cavity walls were thinner in NTM disease than in TB (6.9±3.7mm vs. 10.9±5.6mm, *P*<0.001; 2.8±1.2mm vs. 3.4±1.8mm, *P* = 0.003) (Figs 2 and 3). The ratio of the thickest to the thinnest portion, representing the irregularity of the cavity lesion, was lower in NTM disease than TB (2.6±1.4 vs. 3.7±2.1, *P*<0.001), and thickening of the pleura next to target cavity was more common in NTM disease (80/128 patients [62.5%] vs. 32/128 [25%], *P*<0.001) (Fig 4). In the sub-analysis of the cavity characteristics in which we excluded patients with uncontrolled DM, the results were the same as the results from the whole patient population (See S1 Table).

**Table 2:** CT findings of 256 patients with nontuberculous mycobacterial pulmonary infections and *Mycobacterial tuberculosis* infections.

CT finding^a^		NTM (%) (n = 128)	TB (%) (n = 128)	P-value
**Representative cavity**				
Size (mm)^b^		33±16	41±61	0.163
Location (%)				0.178
	RUL	52 (41)	52 (41)	
	RML	6 (4.7)	1 (0.8)	
	RLL	27 (21)	22 (17)	
	LUL	28 (22)	32 (25)	
	Lingular division	4 (3)	0 (0)	
	LLL	11 (9)	21 (16)	
	Both upper lobes	80 (63)	84 (66)	0.602
Thickness of the thickest cavity wall (mm, mean±SD)^b^		6.9±4	10.9±6	<0.001
Thickness of the thinnest cavity wall (mm, mean±SD)^b^		2.8±1	3.4±2	0.003
The ratio of thickness^b^		2.6±1	3.7±2	<0.001
Thickening of adjacent pleura (%)		80 (63)	32 (25)	<0.001
**Ancillary finding**				
Satellite tree-in-bud pattern nodules, size (%)				<0.001
	None	1 (0.8)	5 (4)	
	<3 mm	79 (62)	15 (12)	
	3–6 mm	31 (24)	50 (39)	
	>6mm	17 (13)	58 (45)	
Satellite tree-in-bud pattern nodules, margin (%)				<0.001
	Ill-defined	92 (72)	21 (17)	
	Well-defined	36 (28)	103 (83)	
	Pleural effusion (%)	5 (4)	4 (3)	0.749
	Number of non-cavitary nodules (≥10mm)^b^	1.0±2	1.7±2	0.007
Bronchiectasis (%)				
	RUL	65 (51)	11 (9)	<0.001
	RML	59 (46)	10 (8)	<0.001
	RLL	50 (39)	10 (8)	<0.001
	LUL	41 (32)	10 (8)	<0.001
	Lingular division	46 (36)	9 (7)	<0.001
	LLL	27 (21)	10 (8)	0.003
	Bronchiectasis involving > 5 lobes (%)	19 (15)	1 (0.8)	<0.001

NTM = Nontuberculous mycobacteria, TB = *Mycobacterium tuberculosis* infection, SD = standard deviation, RUL = right upper lobe, RML = right middle lobe, RLL = right lower lobe, LUL = left upper lobe, LLL = left lower lobe.

^a^ chi-square test

^b^ Student’s t-test

**Fig 5:**
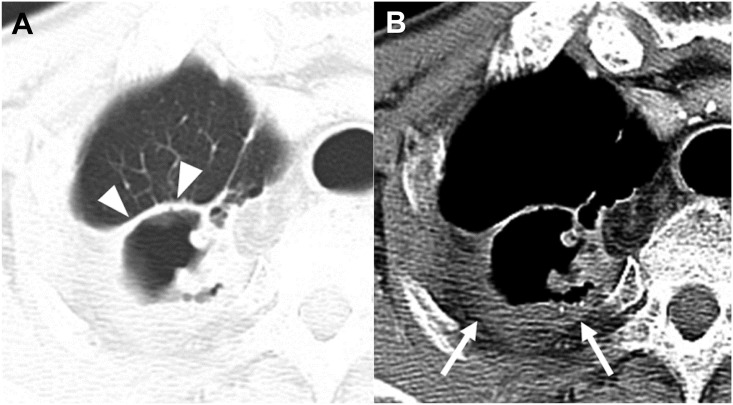
CT images of a *Mycobacterium intracellulare* pulmonary infection in a 77-year-old man. Chest CT axial images were obtained at the level of the right bracheocephalic vein (2.5- mm slice). The lung window setting shows the thin and even thickness of the cavity (arrowheads) in the right upper lobe (**A**). The mediastinal window setting shows thick pleural thickening with proliferation of extra-pleural fat next to the cavity in the right upper lobe (arrows) (**B**).

**Fig 6:**
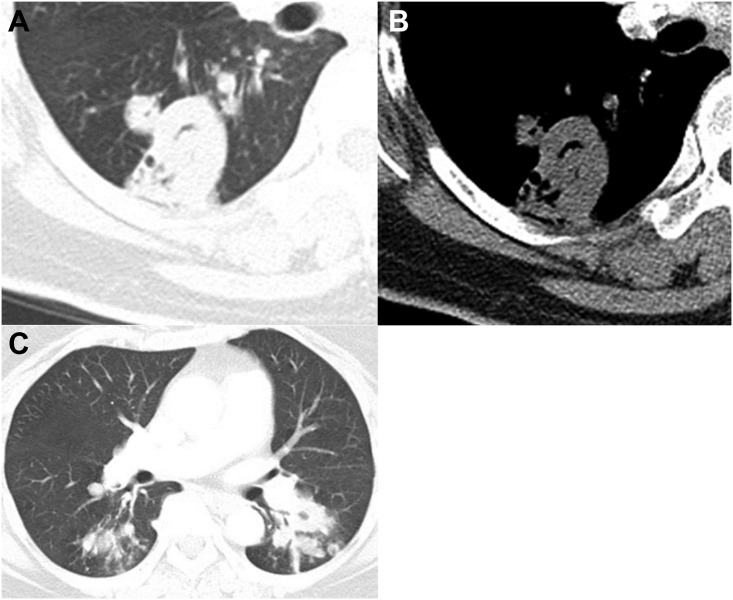
CT images of a *Mycobacterium tuberculosis* infection in a 73-year-old female. Chest CT axial images (2.5 mm collimation) were obtained of the bronchus intermedius (**A, B**) and superior segmental bronchi of both lower lobes (**C**). The lung and mediastinal window settings show a thick-walled cavity in the superior segment of the right lower lobe (**A**) without marked pleural thickening next to the cavity (**B**). (**C**) Note the multiple satellite tree-in-bud nodules in the superior segment of both lower lobes (> 6 mm) and the macronodules in the left lower lobe (≥10mm).

With respect to ancillary findings, satellite tree-in-bud pattern nodules surrounding cavities < 3 mm with ill-defined margins were more common in NTM disease than in TB (*P*<0.001) (Figs 5 and 6). At the same time there were fewer non-cavitary macro-nodules (≥10mm) in NTM disease than in TB (P = 0.007). Bronchiectasis in both lungs was more frequent in NTM disease than TB (all P<0.001 except for LLL [P = 0.003) as was bronchiectasis involvement of > 5 lobes (P<0.001).

In the univariate analysis, the thickness of the thickest cavity wall (OR, 0.96; CI, 0.94–0.99; P = 0.002), the ratio of thickness (OR, 0.92; CI, 0.86–0.99; P = 0.022), thickening of adjacent pleura (OR, 2.5; CI, 1.66–3.77; P<0.001), tree-in-bud pattern nodules <3 mm (OR, 4; CI, 2.45–6.53; P<0.001), ill-defined tree-in-bud nodules (OR, 4.38; CI, 2.73–7.04; P<0.001), number of non-cavitary nodules (≥10mm) (OR, 0.88; CI, 0.79–0.98; P = 0.025), bronchiectasis in all lobes (RUL: OR, 5.91; CI, 3.12–11.2; P<0.001, RML: OR, 5.9; CI, 3.02–11.5; P<0.001, RLL: OR, 5; CI, 2.54–9.86; P<0.001, LUL: OR, 4.1; CI, 2.05–8.19; P<0.001, lingular division: OR, 5.11; CI, 2.5–10.4; P<0.001, and LLL: OR, 2.7; CI, 1.31–5.58; P = 0.007), and the presence of bronchiectasis involving more than 5 lobes (OR, 19; CI, 2.54–141.9; P = 0.004) were associated with NTM disease.

Multivariate analysis logistic regression was calculated for the variables which were significantly associated with NTM disease in the univariate analysis. The thickening of adjacent pleura (OR, 5.54; CI, 2.02–15.2; P = 0.001), ill-defined tree-in-bud nodules (OR, 8; CI, 2.1–30.4; P = 0.002), number of non-cavitary nodules (≥10mm) (OR, 0.72; CI, 0.56–0.9; P = 0.015), and bronchiectasis in RUL (OR, 4.7; CI, 1.3–16.4; P = 0.015) were significantly associated with NTM disease. The results of univariate and multivariate analysis to identify CT findings and also clinical findings in predicting NTM disease are shown in Table 3.

**Table 3:** Results of univariate and multivariate analysis.

		Univariate analysis			Multivariate analysis		
Variable		OR	95% CI	P value	OR	95% CI	P value
**Representative cavity**							
Thickness of the thickest cavity wall (mm)		0.96	0.94–0.99	0.002			
Thickness of the thinnest cavity wall (mm)		0.95	0.89–1.02	0.190			
The ratio of thickness		0.92	0.86–0.99	0.022			
Thickening of adjacent pleura		2.5	1.65–3.78	0.001	5.54	2.02–15.2	0.001
**Ancillary finding**							
Satellite tree-in-bud pattern nodules (referent to >6 mm)							
	<3 mm	4	2.45–6.53	<0.001			
	3–6 mm	0.62	0.4–0.97	0.037			
Margin of tree-in-bud pattern nodules (referent to well-defined)							
	Ill-defined	4.38	2.73–7.04	<0.001	8	2.1–30.4	0.002
	Number of non-cavitary nodules (≥10mm)	0.88	0.79–0.98	0.025	0.72	0.56–0.9	0.015
Bronchiectasis							
	RUL	5.91	3.12–11.2	<0.001	4.7	1.3–16.4	0.015
	RML	5.9	3.02–11.5	<0.001			
	RLL	5	2.54–9.86	<0.001			
	LUL	4.1	2.05–8.19	<0.001			
	Lingular division	5.11	2.5–10.4	<0.001			
	LLL	2.7	1.31–5.58	0.007			
	Bronchiectasis involving > 5 lobes	19	2.54–141.9	0.004			
**Clinical finding**							
BMI		0.99	0.98–1	0.343			
Smoking (reference to current smoker)							
	Ex-smoker	1.13	0.73–1.74	0.583			
	Never smoker	1.75	1.19–2.58	0.005	6.52	1.88–22.6	0.003
	Previous TB history	3.65	2.13–6.24	<0.001	3.57	1.23–10.4	0.02
Underlying disease							
	DM	0.3	0.17–0.55	<0.001			
Symptom							
	Cough	1.3	0.98–1.74	0.07			
	Sputum	1.32	0.96–1.8	0.083			
	Hemoptysis	2.19	1.21–3.95	0.009			
	Dyspnea	0.05	0.01–0.39	0.004	0.05	0.003–0.74	0.03

OR = odds ratio, CI = confidence interval.

## Discussion

We have reported CT findings in NTM disease and compared them with those in TB, focusing on the largest and most representative cavities and associated ancillary findings. Our results revealed the characteristics of the cavities in NTM disease, showing that they were thinner and more even walled than those seen in TB. However, in multivariate analysis, thickening of the pleura adjacent to cavities was the only significant factor of the representative cavity findings in the multivariate analysis.

There have been reports of some differences between TB and NTM in the course of cavity formation, which could explain the different characteristics of the cavities in NTM disease and TB in our study: The cavity in TB is known to be formed by the coalescence of several tiny centrilobular cavities as the disease progresses [20,21], and in the stage of active caseous air-space consolidation, the cavity wall is usually thick and has an indistinct outer margin [17]. And these reports support our study results of relatively thicker and irregular walls of TB. For NTM disease, the previous study suggested that NTM disease begins with bronchial wall thickening, followed by peribronchial thickening or nodules, and develops into inflamed cystic bronchiectasis that manifests as cavities [22]. Therefore, we believe this study’s results also support the results of our study, in which we observed thinner and more even cavity walls in NTM.

It is possible that the thickness and sharpness of the cavity walls are dependent on the stage of the disease; however, the proportion of patients with a positive sputum AFB smear was similar between NTM and TB in our study, which could be used as a marker of disease activity. Additionally, WBC counts and CRP level, which were used as inflammatory biomarkers for disease activity in TB or NTM in previous studies [23,24], were not significantly different between TB and NTM disease.

Pleural thickening adjacent to the cavity was the most significant predictor for NTM disease in our study, but only few previous studies had been reported this finding [12]. Christensen et al. reported that thickening of the pleura immediately adjacent to cavities occurred in both NTM and TB, but it was more common with NTM disease than TB (56% of *M*. *intracellularis*, 37% of *M*. *kansasii*, and 24% of TB) [12,25]. In addition, extensive pleural thickening > 2 cm was more commonly observed in NTM disease than TB (16% of *M*. *intracellularis*, 3% of *M*. *kansasii*, and none of TB). This finding was thought to be due to a more indolent and chronic course of NTM disease. However, because these studies were performed using only chest radiographs, it was not possible to discern how much of pleural thickening was truly pleural and represented parenchymal disease in the wall of adjacent cavity.

Previously, although there have been several studies describing the characteristics of the cavities in NTM disease using radiographs or CT, there were no clear-cut differentiating features. Some studies reported that NTM disease tended to cause smaller and thinner-walled cavities than TB [12,16,25,26], but at least one found no difference in cavity wall thickness [27]. Furthermore, Moore et al. reported the presence of relatively thick-walled cavities in CT images of NTM disease [14]. However, we believe these previous studies have significant limitations: because they were performed in the 1980’-1990’s some used only chest radiographs, and the quality of the radiographs and CT images in these early studies must have been poorer than ours.

Our analysis of the ancillary findings revealed that smaller satellite tree-in-bud pattern nodules (< 3 mm), satellite nodules with ill-defined margin, fewer non-cavitary nodules of ≥10 mm, bronchiectasis in all lobes, and the presence of bronchiectasis involving > 5 lobes were more frequent in NTM disease than TB. Also, ill-defined nodule margins and rare non-cavitary macronodules (≥ 10 mm) were significant factors predicting NTM disease. In TB, bronchogenic dissemination is the most common route of spreading [21,28], and manifests as multiple, 5- to 10-mm nodules with segmental or lobar distributions [28]. NTM disease and TB are similar in terms of bronchogenic spreading, but the former has a more chronic, indolent course than TB. Microscopically there was also sloughing of the epithelial lining and circumferential replacement of the bronchial wall architecture by granulomatous inflammation [29]. Such differences in the patterns of progression of the two types of infection might be responsible for the differences in CT findings.

Radiologists as well as pulmonologists are generally unable to distinguish between NTM disease and TB, particularly in patients with dominant cavities, because of the considerable overlap in the clinical features of the two diseases [30]. In our study, symptoms such as cough, sputum, and hemoptysis were significantly more frequent in NTM disease and dyspnea was significantly more frequent in TB. However, we believe that these symptoms would not help clinicians distinguish between NTM disease and TB, because they are quite nonspecific.

In our study, there were 60 patients with DM, and they were significantly more numerous among the TB patients than among those with NTM disease. In previous reports, the presence of cavities was associated with HbA1C level, and cavity formation was significantly more common in uncontrolled DM patients than in non-DM patients [19,31]. Also, whereas a previous study showed no significant difference in the formation of cavities between non-DM and well-controlled DM in TB patients [19], there has yet to be any corresponding reports on NTM. In our study, there was no significant difference in the status of diabetes control among DM patients in NTM and TB patients. Considering the unknown effects of DM on cavity characteristics, we excluded patients with uncontrolled DM in the sub-analysis, which yielded no differences in comparison to the results of the whole study population. Therefore, uncontrolled DM may affect cavity formation in TB or NTM disease, but it may not affect the characteristics of the cavities. We believe further study is needed on this subject in order to clearly define the relationship between DM and cavities.

Because we matched age and gender in the patient selection process, there were no significant difference in age and gender between the patients with NTM disease and TB in our study. However, in previous studies, NTM disease was more frequent in older patients and postmenopausal women than TB [32–36].

This study had some limitations. First, it was a retrospective study in a single center in a country with semi-endemic TB. Therefore, some clinical characteristics may have been under- or over-estimated. Second, it included different species of NTM, which might have influenced the characteristics of the lesions, e.g. it has been reported that 84% of infective foci of *M*. *kansasii* had cavities [12,25]. In our study, only 6.3% of patients who had *M*. *kansasii*, and the most common pathogen was *M*. *intracellulare*. Therefore, further studies are necessary to compare the imaging characteristics of TB and *M*. *kansasii* in a larger population. Third, there were several confounding factors in patient selection such as history of TB, COPD, and DM which could not be completely excluded all.

In conclusion, the major cavities in NTM disease generally have thinner and more even walls than those in TB. When cavities are associated with adjacent pleural thickening, ill-defined satellite tree-in-bud nodules, or fewer non-cavitary nodules ≥10 mm, these CT findings are highly suggestive of NTM disease rather than TB.

## Supporting information

S1 Table CT findings of 256 original patients and 232 patients excluding uncontrolled DM with nontuberculous mycobacterial pulmonary infections and *Mycobacterial tuberculosis* infections. (DOCX)

S1 Appendix Minimized dataset of the study. (XLSX)

## Abbreviations

AUC: area under the curve
BMI: body mass index
COPD: chronic obstructive pulmonary disease
HIV: human immunodeficiency virus
LLL: left lower lobe
LUL: left upper lobe
MAC: *Mycobacterium avium-intracellulare complex*
NTM: nontuberculous mycobacteria
RLL: right lower lobe
RML: right middle lobe
ROC: receiver operating characteristic
RUL: right upper lobe
TB: *mycobacterium tuberculosis* infection
