# Supplementary material for: Comparison of chest CT findings in nontuberculous mycobacterial diseases vs. Mycobacterium tuberculosis lung disease in HIV-negative patients with cavities
Source: PLoS One. 2017 Mar 27;12(3):e0174240. doi: 10.1371/journal.pone.0174240 (PMC5367717; doi:10.1371/journal.pone.0174240)
Supplement: S1 Table — (DOCX) [file pone.0174240.s001.docx]

**Supporting information**

**Table S1. CT findings of 256 original patients and 232 patients excluding uncontrolled DM with nontuberculous mycobacterial pulmonary infections and *Mycobacterial tuberculosis* infections.**

| **CT findings** | **Original results** | | | **Excluding uncontrolled DM** | | |
| --- | --- | --- | --- | --- | --- | --- |
|  | **NTM (%) (n=128)** | **TB (%) (n=128)** | **P-value** | **NTM (%) (n=121)** | **TB (%) (n=111)** | **P-value** |
| Size (mm) | 33±16 | 41±61 | 0.163 | 33±16 | 41±65 | 0.169 |
| Thickness of the thickest cavity wall (mm, mean±SD) | 6.9±4 | 10.9±6 | <0.001 | 6.7±4 | 10.5±6 | <0.001 |
| Thickness of the thinnest cavity wall (mm, mean±SD) | 2.8±1 | 3.4±2 | 0.003 | 2.8±1 | 3.4±2 | 0.008 |
| The ratio of thickness | 2.6±1 | 3.7±2 | <0.001 | 2.6±1 | 3.6±2 | <0.001 |
